# Impact of COVID-19 pandemic in natural course of Moyamoya Angiopathy: an experience from tertiary-care-center in India

**DOI:** 10.1186/s41983-021-00412-2

**Published:** 2021-12-11

**Authors:** Shambaditya Das, Biman Kanti Ray, Ritwik Ghosh, Samya Sengupta, Alak Pandit, Souvik Dubey

**Affiliations:** 1 Department of Neurology, Bangur Institute of Neurosciences, IPGMER and SSKM Hospital, 52 1/A Sambhu Nath Pandit Road, Bhowanipore, Kolkata, West Bengal 700020 India; 2 Department of General Medicine, Burdwan Medical College and Hospital, Burdwan, West Bengal India; 3 Department of General Medicine, Apollo Gleneagles Hospital, Kolkata, West Bengal India

**Keywords:** Moyamoya angiopathy, COVID-19, SARS-CoV-2, TIA in Moyamoya angiopathy, Moyamoya angiopathy in COVID-19

## Abstract

**Background:**

COVID-19 mediated immune dysregulation and cytokine storm can precipitate and aggravate Moyamoya angiopathy (MMA), influencing its disease course. This index study was undertaken to prospectively evaluate the status of neurological symptoms of MMA in relation to COVID-19 affection.

**Methodology and results:**

Follow-up MMA patients of institute’s Stroke-clinic were telephonically interview from 24th March to 30th September, 2020. The first call familiarized them with COVID-19 symptoms and neurological manifestations of MMA, followed by monthly-calls with predesigned questionnaire. Patients with suggestion of COVID-19 underwent nasopharyngeal-swab-testing for COVID-19 Reverse transcription-polymerase chain reaction (RT-PCR) positive cases were subjected to antibody levels for COVID-19 Enzyme-linked immunoassay (ELISA) 8–12 weeks after recovery. During symptomatic phase till 14 days of asymptomatic, they were contacted daily/alternate day. Any new onset/worsening of neurological symptoms were noted. The baseline clinico-radiological details were obtained from stroke-clinic registery. Subsequently, all data were analyzed and compared using descriptive statistics. Seventy four of 104 MMA patients could be contacted and enrolled. The mean age, time since last follow-up and compliance to previously prescribed medication were 23.5 ± 16.1 years, 9.2 ± 1.7 months and 90.5% (*n* = 67), respectively. Aggravation/new onset neurological symptom were seen in 64.3% (*n* = 9) of COVID-19 positive MMA (*n* = 14), of which 8 were seen among the 11 pediatric COVID-19 positive MMA [(Transient ischemic attacks) TIA-4, TIA with headache-1, seizure-2, stroke causing mortality-1].

**Conclusion:**

COVID-19 infection can potentiate MMA causing significant morbidity and mortality, especially in children. Providing optimal care for severe diseases (such as MMA) in developing countries during pandemic remains a challenge.

## Background

Moyamoya angiopathy (MMA) is an intracranial angiopathy of unknown etiology, characterized by bilateral (B/L) progressive steno-occlusive changes of the intracranial portion of the internal carotid arteries (ICA) and proximal portions of the anterior cerebral artery (ACA) and/or middle cerebral artery (MCA) with development of compensatory abnormal vascular network at the base of the brain appearing as “puff of smoke”, frequently complicated by wide array of recurrent neurological symptoms [1–3]. Inflammatory state has been postulated to initiate and enhance progression of changes of MMA influencing the cytokine pathways [4]. Besides, hemodynamic stress related to infection and raised body temperature has been known to precipitate symptoms of MMA [5, 6].

The current severe acute respiratory syndrome coronavirus 2 (SARS-CoV-2) pandemic which was declared a public health emergency of international concern on January 2020 has been known to cause immune dysregulation and syndrome of cytokine storm [7, 8]. Thus being a potential condition to influence the disease process of MMA, aside from an independent risk factor for stroke [6, 8]. To contain the epidemic there were also widespread “lockdowns” in India, resulting in difficulty in getting access to health care facilities which lead to the initiation of “teleconsultation” services [9]. A precise representation of the status of patients of MMA in this SARS-CoV-2 pandemic is lacking. This is the first systematic study to evaluate the status of neurological symptoms in relation to COVID affection in a cohort of MMA patients from an area of high disease load of eastern India by telephonic survey.

## Methods

A prospective study was undertaken between the time period of 24th March, 2020 (the day lockdown was declared in India) and 30th September, 2020 among the patients previously diagnosed as MMA by angiographic studies and who has been under the regular follow-up of Stroke-clinic of Bangur Institute of Neurosciences, Institute of Post-Graduate Medical Education and Research, Kolkata, India. An attempt was made to contact via telephone every MMA patient under our follow-up, during COVID-19 pandemic and a verbal consent was taken from the patient or patient’s kin (in case of minors less than 18 years of age or patients with cognitive impairments that incapacitates them from giving consent). The method of telephonic interview was chosen due to its feasibility in the times of COVID-19 pandemic because of the lockdown and the imposed travel restrictions which made physical follow-up a difficult proposition. The first phone call was made on 24th and 25th March, 2020 and every contacted MMA patient or patient’s kin were familiarized with the possible symptoms of COVID (fever, sore throat, dry cough, dyspnea, pain abdomen, diarrhea, loss of smell, loss of taste, headache, red eyes, myalgia, skin rashes) and the neurological manifestations of MMA (fixed motor weakness, headache, seizure, transient ischemic attack, visual symptoms and extra-pyramidal symptoms), explained in a simplified way and detailed manner in patient’s own language. The baseline-data of every enrolled MMA patient was derived using predesigned sets of questions that included demographic details, duration of disease, compliance to prescribed medications, time since last follow-up, status of neurological symptoms—any new onset or worsening of previous symptom, co-morbidities, history of contact with confirmed COVID-19 patients, involvement of family members, travel history, and presence of symptoms suggestive of COVID-19. They were made aware of safety practice during this COVID-19 pandemic, such as social distancing and personal hygiene. They were also provided our contacts so that they can reach out to us at any time if required. The details of “MMA” type, characterization of brain lesion in imaging, suzuki staging [10] were obtained from the records of last visit of the patient to Stroke-clinic of Bangur Institute of Neurosciences. Following the first telephonic interview, every enrolled patient was contacted at monthly intervals and using pre-designed sets of questions were probed about status of neurological symptoms, history of contact with confirmed COVID-19 patients, involvement of family members, travel history, and presence of symptoms suggestive of COVID-19. Those patients who reported symptoms suggestive of COVID-19 were advised to undergo nasopharyngeal swab testing for COVID-19 RT-PCR and to seek help in nearby health-care facility designated for COVID-care and they were telephonically reached out to several times (daily or alternate day) during the symptomatic phase till 14 days after resolution of symptoms or till mortality and directed interview to any new onset or worsening of neurological symptoms during that period were done to keep a tab on the clinical course and guidance for further medical attention. Those who tested positive for COVID-19 RT-PCR nasopharyngeal swab testing, were further subjected to antibody levels for COVID-19 (ELISA method) 8–12 weeks after recovery.

SPSS version 25.0 (year—2021, manufacturer—International Business Machines Corporation, country of origin—USA) was used for statistical analysis. Data were summarized by routine descriptive statistics, mean and standard deviation for numerical variables that are normally distributed, median and inter-quartile range (IQR) for skewed numerical variables and counts and percentages for categorical variables. Comparison between two groups of numerical variables was done by Student’s independent samples *t* test, if normally distributed, or by Mann–Whitney *U* test, if otherwise. Fischer’s exact test or Pearson’s Chi-square test were used for intergroup comparisons of categorical variables. Analyses were two-tailed and statistical significance level was set at *p* < 0.05 for all comparisons.

## Results

Whole cohort—Among the 104 MMA patients under the follow-up of Stroke-clinic of Bangur Institute of Neurosciences, Institute of Post-Graduate Medical Education and Research, Kolkata, India, 74 patients could be contacted over telephone and were enrolled for the study. The mean time since last follow-up was 9.2 ± 1.7 months. The compliance to previously prescribed medication was noted in 90.5% (*n* = 67), while worsening or new onset neurological symptom was seen in 12 patients (16.2%) (Table 1).

**Table 1:** Demographic, clinical, radiological and follow-up of entire cohort and suspected or confimed COVID-19 cohort

	Entire cohort (*n* = 74)	Suspected or confirmed COVID-19 cohort (*n* = 14)
1. Mean age (years)	23.5 ± 16.1	17.6 ± 16.1
2. Pediatric MMA	52.7%	78.6%
3. Adult MMA	47.3%	21.4%
4. Male:Female	1:1.24	1:1
5. Area of residence		
Rural	64.9%	71.4%
Urban	35.1%	28.6%
6. Duration of disease (months)	41.4 ± 31.5	36.3 ± 12.9
7. “MMA” type		
TIA	2.7%	7.1%
Frequent TIA	16.2%	35.7%
Cerebral infarction	62.2%	57.1%
Intracerebral hemorrhage	8.1%	0%
Headache	5.4%	0%
Epilepsy	5.4%	0%
Asymptomatic	0%	0%
Others	0%	0%
8. Previously documented radiological lesion in brain		
Infarct	85.1%	78.6%
Hemorrhage	6.8%	0%
No acute lesion	8.1%	21.4%
9. Suzuki staging in the last documented in angiography		
Stage I	0%	0%
Stage II	9.5%	7.1%
Stage III	29.7%	28.6%
Stage IV	37.8%	50.0%
Stage V	21.6%	14.2%
Stage VI	1.4%	0%
10. Time since last follow-up (months)	9.2 ± 1.7	9.6 ± 1.6
11. Compliance to prescribed medications		
Yes	90.5%	92.9%
No	9.5%	7.1%
12. Neurological symptoms (worsening or new onset)		
Yes	16.2%	64.3%
No	83.8%	35.7%

*n*: Number; MMA: Moyamoya angiopathy; TIA: Transient ischemic attack

Suspected and confirmed COVID-19 cohort—Symptoms suggestive of COVID-19 were seen in 14 of the 74 contacted patients of MMA. Among the 14 patients, 12 had undergone nasopharyngeal swab testing for COVID-19 RT-PCR and all of them were tested positive, while rest 2 did not undergo testing despite symptoms. However, all the 14 patients underwent IgG antibody testing for COVID-19 (ELISA) at 10–12 week interval following resolution of COVID-19 symptoms, and all of them were positively confirmed with high IgG titres. Of them 10 patients (71.4%) had history of (h/o) contact with COVID-19 patient with close temporal association with onset of flu-like symptoms in them. Only 1 patient, tested and confirmed with positive COVID-19 RT-PCR, was hospitalized later on with acute onset right sided hemiparesis then altered sensorium followed by death. The rest of them did not require hospitalization and managed at home-quarantine. They were mostly of rural residence, representing 71.4% (*n* = 10) of patients; however, all of them had their residence in containment zone (*n* = 14). The mean time since last follow-up was 9.6 ± 1.6 months. Thirteen patients (92.9%) were compliant on medications.

After an onset of flu-like illness, nine patients (64.3%) demonstrated aggravation or appearance of new onset neurological symptoms. Eight of them were among the 11 pediatric MMA patients; 5 had increased frequency of TIA (1 of them had associated aggravation of headache symptoms), 2 had episodes of seizure (1 patient among them was non-compliant on prescribed medications) and 1 had fixed motor weakness and finally succumbing to his ailment (Table 2).

**Table 2:** Summarization of neurological status and details pertaining to COVID-19 symptomatology and behavior of suspected or confirmed COVID-19 cohort

	Child (*n* = 11) (%)	Adult (*n* = 3) (%)	Total (*n* = 14) (%)
1. Symptomatology related to COVID-19			
(a) Fever	100	100	100
(b) Others			
I. Sore throat	90.9	66.7	85.7
II. Dry cough	45.5	100	57.1
III. Dyspnea	0	0	0
IV. Pain abdomen	9.1	0	7.1
V. Diarrhea	27.3	33.3	28.6
VI. Loss of smell	36.4	100	50.0
VII. Loss of taste	18.2	100	35.7
VIII. Headache	27.3	100	42.9
IX. Red eyes	0	0	0
X. Myalgia	81.8	100	88.9
XI. Skin rashes	0	0	0
2. Involvement of family members	63.6	66.7	64.4
3. History of contact with COVID-19 patients	63.6	100	71.4
4. Travel history	0	66.7	14.3
5. Hospitalization	9.1	0	7.1
6. Residence in containment zone	100	100	100
7. Co-morbidities			
(a) Hypertension	0	66.7	14.3
(b) Type II Diabetes Mellitus	0	33.3	7.1
(c) Hypothyroidism	0	0	0
(d) Renal dysfunction	0	0	0
(e) Cardiac dysfunction	0	0	0
(f) Liver dysfunction	0	0	0
(g) Hematological dysfunction	9.1	0	7.1
8. Neurological symptom (worsening or new onset)			
(a) Fixed motor weakness	9.1	0	7.1
(b) Seizure	18.2	0	14.3
(c) Headache	9.1	33.3	14.3
(d) Transient ischemic attack	45.5	0	35.7
(e) Cognitive and behavioral symptoms	0	0	0
(f) Visual symptoms	0	0	0
(g) Extra-pyamidal symptoms	0	0	0

*n*: Number

## Discussion

Moyamoya Angiopathy is known to be a form of immune reactive Vasculitis resulting from vascular immune injury and inflammation response [11, 12]. Inflammation promotes onset or progression of MMA via two major mechanism: (I) anti-inflammatory mediators (IL-4, IL-10, IL-13, IFN-α and TGF-β) affecting vascular reactivity and auto-regulation leading to acceleration or acute aggravation of MMA, (II) pro-inflammatory mediators (IFN-β, IFN-γ, TNF-α, IL-1, IL-6) activating RNF dependent signal transducing pathway and influencing initiation and often fulminant progression of MMA. T-cell mediated autoimmune response can also accelerate the pathology. Thus, an acute systemic inflammatory state is anticipated to cause hyperplasia of the intimal vascular smooth muscle cells and neovascularization by proliferation of endothelial cells, leading to angiogenesis heralding luminal narrowing and collateral formation. In the same lines, SARS-CoV2 induced “cytokine storm” with increase in inflammatory cytokine may influence RNF-213 and caveolin-1 leading to onset and progression of MMA [4, 6].

Besides many factors including dehydration, systemic hypotension, recent infection, hot bath, vigorous exercise, emotional outburst, increased body temperature, crying or hyperventilation can potentiate precipitation of symptoms of MMA by causing transient cerebral hypoperfusion. Infection with SARS-CoV2 and associated hemodynamic stress with emotional disruption and cytokine storm could act as precipitating factors for MMA [5, 6].

The risk of ischemic CVA by itself is more with COVID-19 infection compared to influenza [13]. The three important mechanisms implicated are hypercoagulability, vasculitis and cardiomyopathy. The viral invasion of the vascular endothelium leads to activation of contact and complement system which further initiates inflammatory cascades. The affinity of COVID-19 to the ACE2 receptors expressed abundantly on vascular endothelium, leads to both direct local as well as systemic immune response to pathogen (“cytokine storm”) leading to a widespread thrombosis, microangiopathy and angiogenesis, which disrupts the ACE2 mediated regulation of sympathoadrenal system, vascular autoregulation and cerebral blood flow [8, 14].

Among the 74 patients who could be contacted, 14 patients had features of COVID-19 symptoms, 9 of them suffered from aggravation or new onset neurological symptoms, 8 of them were of pediatric age group. Among the pediatric MMA, only one patient suffered from a new onset fixed motor paresis and later on died most likely by its complications. While the other 7 patients had aggravation of their previous neurological symptom, 5 of them had increase in frequency of TIA and 2 of them suffered from breakthrough seizure. The only adult patient with symptoms suggestive of COVID-19 and aggravation of neurological symptoms had worsening of headache frequency and severity.

The aggravation of TIA symptoms were clinically more significant than occurrence of breakthrough seizures, since focal motor seizure often are residual signs independent from the perfusion status, whereas TIA represents hemodynamic insufficiency which probably precipitated in the background of hemodynamic stress and SARS-CoV2 infection [5, 6, 15]. Moreover, in MMA, it may be extremely difficult to interpret ischemic events, since many MRI-evident ischemic lesions can be clinically silent, while clinical TIA often can be associated with cerebral infarction on brain imaging [16]. Thus, patients with aggravation of TIA in our cohort following flu-like symptoms may have sustained permanent tissue damage of brain, which can be confirmed by brain imaging only after physical follow-up.

The worsening of headache during and immediately after COVID-19 infection can be related to the aggravated cerebral hypoperfusion or may be part of TIA symptom by itself. The already chronically hypoperfused cerebral circulation in patients of MMA leads to lowering of migraine threshold and the ischemic penumbra in states of aggravated hypoperfusion can result in increased risk of spreading cortical depression resulting in migrainous aura [6, 17].

Besides the 2 patients with COVID-19 infection, another 2 patients without any symptoms suggestive of COVID, also of pediatric age group, had suffered breakthrough seizure and non-compliance on anti-epileptic drugs was noted in 3 from these 4 patients. The prevailing COVID-19 pandemic had led to strict travel restrictions, and since majority of our patients were of rural residence, the formation of containment zone according to government policy and curtailment of public transport had greatly deterred the regular physical follow-up of those patients [9], leading to a protracted period of time since last follow-up of 9.2 ± 1.7 months. In addition, a decreased financial capacity to buy the medications on their own or unavailability for the previously prescribed drugs in their locality has led to non-compliance in 9.5% of our population, leading to the risks of breakthrough seizure.

The major limitation of our study included (1) inability to search out to a major fraction of MMA patients who were a regular follow-up of our stroke-clinic previously, (2) no physical follow-up was possible in the study period, and (3) brain imaging could not be done in our facility even in patients with aggravation of neurological symptoms. However, strength of the study remains in its very frequent verbal contact with a relatively large population of patients harboring a rare disease such as MMA during COVID-19 pandemic allowing the understanding the impact of later on the former.

## Conclusion

Our study was the first detailed survey regarding the impact of COVID-19 on Moyamoya patients and the status of their neurological symptoms, from an area of high transmission and case load, comprising of the largest cohort of MMA in India. COVID-19 infections can potentiate MMA symptoms predominantly related to critical hemodynamic compromise and can lead to severe morbidity and mortality, especially in children. Besides, the imposition of social restrictions has led to a great challenge in providing medical care for patients with severe disease such as MMA, culminating in non-compliance to medications and deferred routine follow-up.

## Data Availability

The data that support the findings of this study are available from the corresponding author upon reasonable request.

## Abbreviations

MMA: Moyamoya angiopathy
B/L: Bilateral
ICA: Internal carotid arteries
ACA: Anterior cerebral artery
MCA: Middle cerebral artery
SARS-CoV-2: Severe acute respiratory syndrome coronavirus 2
RT-PCR: Reverse transcription-polymerase chain reaction
ELISA: Enzyme-linked immunoassay
IQR: Inter-quartile range
M:F: Male:female
TIA: Transient ischemic attacks
MRI: Magnetic resonance imaging
h/o: History of
IL: Interleukin
IFN-α: Interferon alpha
TGF-β: Transforming growth factor beta
IFN-β: Interferon beta
IFN-γ: Interferon gamma
TNF-α: Tumour necrosis factor alpha
RNF-213: Ring finger protein 213
ACE2: Angiotensin converting enzyme 2
